# Identifying competencies for integrated knowledge translation: a Delphi study

**DOI:** 10.1186/s12913-021-07107-7

**Published:** 2021-10-30

**Authors:** Euson Yeung, Stephanie Scodras, Nancy M. Salbach, Anita Kothari, Ian D. Graham

**Affiliations:** 1 University of Toronto, 160-500 University Avenue, Toronto, Ontario M5G 1V7 Canada; 2 Western University, Health Sciences Building, Rm 222, 1151 Richmond St, London, Ontario N6A 5B9 Canada; 3 School of Epidemiology and Public Health, University of Ottawa, 600 Peter Morand Cresent, Ottawa, Ontario K1G 5Z3 Canada; 4 Centre for Practice-Changing Research, The Ottawa Hospital Research Institute, 501 Smyth Road, Box 711, Ottawa, Ontario K1H 8L6 Canada

**Keywords:** Integrated knowledge translation, Knowledge user, Researcher, Competence, Delphi

## Abstract

**Background:**

Considerable progress has been made to advance the field of knowledge translation to address the knowledge-to-action gap in health care; however, there remains a growing concern that misalignments persist between research being conducted and the issues faced by knowledge users, such as clinicians and health policy makers, who make decisions in the health care context. Integrated knowledge translation (IKT) is a collaborative research model that has shown promise in addressing these concerns. It takes advantage of the unique and shared competencies amongst researchers and knowledge users to ensure relevance of the research process and its outcomes. To date, core competencies have already been identified to facilitate training in knowledge translation more generally but they have yet to be prioritized for IKT more specifically. The primary aim of this study was to recruit a group of researchers and knowledge users to identify and prioritize core competencies for researchers and knowledge users to engage with IKT.

**Methods:**

We recruited health care knowledge users (KUs) and researchers with experience and knowledge of IKT for a quantitative, cross-sectional study. We employed a modified Delphi approach consisting of three e-survey rounds to establish consensus on competencies important to IKT for KUs and researchers based on mean rating of importance and agreement between participants.

**Results:**

Nineteen (73%) of the initial 26 participants were researchers (response rate = 41% in the first round; retention in subsequent rounds > 80%). Participants identified a total of 46 competencies important for IKT (18 competencies for KUs, 28 competencies for researchers) under 3 broad domains. Technical research skills were deemed extremely important for researchers, while both groups require teamwork and knowledge translation skills.

**Conclusions:**

This study provides important insight into distinct and overlapping IKT competencies for KUs and researchers. Future work could focus on how these can be further negotiated and contextualized for a wide range of IKT contexts, projects and teams. Greater attention could also be paid to establishing competencies of the entire team to support the research co-production process.

**Supplementary Information:**

The online version contains supplementary material available at 10.1186/s12913-021-07107-7.

## Background

Over the last few decades, considerable progress has been made to advance the field of knowledge translation to address the knowledge-to-action gap in health care. Among these advances is the increasing recognition that challenges with the uptake of evidence into health care policy and practice are not only attributed to underutilization of research evidence, but may also stem from failures in the knowledge production process. Scholars have voiced a growing concern that misalignments may exist between the research conducted and the issues faced by knowledge users, such as clinicians and policy makers, who make decisions in the health care context [1–4]. These incongruences have led to views that research results are inaccessible and have limited applicability to everyday clinical practice.

Integrated knowledge translation (IKT) is a collaborative research model that has shown promise in addressing the aforementioned views [3, 4]. This approach involves researchers and knowledge users (clinicians, managers, policy makers, etc.) “engaging in a mutually beneficial research project or program of research” wherein knowledge users identify a problem and have the authority to implement the research recommendations [5, 6]. Thus, the IKT approach considers researchers and knowledge users as co-producers of knowledge [7–11].

Research co-production has been described as “a collaborative endeavour of academic and non-academic actors” [12], characterized by “a space where science intersects with non-science” [13]. Academic and non-academic participants in the research endeavour interact throughout the research process to co-produce knowledge rather than only interacting during dissemination of the research results. The nature of such collaborations is sustained for a period of time, and roles and responsibilities between researcher and knowledge user are at times necessarily indistinct. As such, IKT approaches take advantage of the unique as well as shared competencies amongst researchers and knowledge users [14, 15]. Consequently, the knowledge that is co-produced through these collaborative efforts is more likely to be contextually-relevant and therefore applied in health care practice and policy.

Although the ideals of research co-production are not new, conceptualization of the researcher and knowledge user roles within the research process requires greater attention, especially with respect to how capacity can be built to improve and sustain the practice of IKT. For example, since ongoing collaborative relationships between researcher and knowledge user is a key feature of the IKT process [16], it follows that both groups may need to develop competence in forming, contributing, and sustaining collaborative working relationships for the purpose of co-producing research.

A range of enabling conditions have been identified to support organizations to succeed in IKT. Categorized as organizational, professional and individual-level conditions, these constitute a framework by which organizations can develop and monitor their capacity for IKT [17]. Of the individual-level conditions identified, acquisition of adequate IKT-specific knowledge and skills, or competence, were deemed as essential to the IKT process. To date, core competencies have already been identified to facilitate training in knowledge translation more generally [18]; however competencies have yet to be prioritized for research co-production more specifically. What remains to be clarified is the extent to which the competencies identified by Mallidou et al. [18] pertain to either or both researchers and knowledge users.

The primary aim of this study was to recruit a group of researchers and knowledge users to identify and prioritize core competencies for researchers and knowledge users to engage with IKT.

## Methods

We used a modified Delphi approach [19, 20] to identify and prioritize a set of IKT competencies for the two key groups of stakeholders involved in the research process: knowledge users (KU) and researchers. The Delphi technique is an established and widely used methodology in healthcare research that enables consensus building among individuals with pertinent experience and potentially diverse views [21]. Results from Delphi studies are considered data generated at a single point in time for a particular group of individuals, and are therefore intended for further testing and verification to ultimately inform theory and practice [19].

### Participants and recruitment

We considered two stakeholder groups to be relevant in this study: knowledge users (KUs) and researchers who are familiar and experienced with partnered research using an IKT approach. We define a KU as “an individual who is likely to be able to use the knowledge generated through research to make informed decisions about health policies, programs and/or practices” [22]. Knowledge users in the health care system, such as organizational or system-level decision-makers in health care settings (clinical managers, health care managers, policy-makers), and point of care health care providers, were eligible to participate. Individuals who were a KU or researcher and who had worked in the health sector for at least 2 years were considered eligible. Potential study participants were identified through the Integrated Knowledge Translation Research Network (IKTRN), and through the research team’s professional network. The IKTRN is a network of “knowledge users and researchers committed to studying, teaching and practicing integrated knowledge translation” in Canada [23] or are known to the research team to have experience in the field. Given the uniqueness of the patient perspectives with different health conditions and healthcare settings, and the growing focus specifically on patient engagement in health research, we opted to focus this study exclusively on clinician and health system knowledge users and therefore did not include patients as study participants. Since sample sizes for Delphi panels remain variable [19, 24], we prioritized the need for participants to reflect the full range of KU and researcher stakeholders to optimize the credibility and acceptability of the Delphi results [24], with an ultimate pragmatic target sample size of 20–25 panelists.

Following a modified Dillman approach for recruitment [25], we sent a pre-notice email prior to the first invitation to participate in round one of the survey, followed by two thank you/reminder emails and a final reminder email for each survey round. Sending multiple email reminders is an appropriate method of increasing response rates in Delphi studies [26].

### Ethical considerations

Participants demonstrated implied consent by responding to the online questionnaire and provided their email address if they agreed to be contacted in subsequent rounds. The University of Toronto Research Ethics Board approved the study protocol.

### Modified Delphi methodology

Our modified Delphi methodology was established a priori and consisted of three rounds, which is considered to be the optimal number of rounds [21]. We asked participants to rate the importance of competencies needed by KUs and researchers who are engaging in IKT on a five-point ordinal scale (Not Important, Slightly Important, Important, Very Important, Extremely Important) through an online questionnaire developed using SurveyMonkey™. In addition, participants were asked to identify new competencies not already included in the questionnaire. Participant feedback was provided between rounds as this is an important aspect of developing group consensus [27, 28]. In rounds two and three, we provided participant feedback in the form of the overall median rating and the percent of the participants who rated the competency as either ‘Very Important’ or ‘Extremely Important’ from the previous round for each competency being rated. Data were collected over a course of 2 months (April – June, 2019), and participants had 2 weeks to complete each round with 1 week separating the rounds.

#### Identifying the initial list of competencies

The initial list of competencies for KUs and researchers was developed based on the ‘Knowledge Translation Pathways (KTP)’ tool that arose from a scoping review of competencies for knowledge translation [18]. This is in keeping with other Delphi studies that utilized pre-existing information for the initial Delphi round [29]. Two members of the research team (EY, SS) reviewed and identified competencies that were most relevant to IKT. EY is an Integrated Knowledge Translation Expert member of the IKTRN and SS is a healthcare professional and research trainee with graduate level education in knowledge translation. They synthesized the list into 29 competencies for KUs and 30 competencies for researchers through discussion. In keeping with the original ‘KTP’ tool, the competencies were divided into domains of ‘Evidence’, ‘Teamwork’, and ‘Knowledge Translation (KT) Activities’ rather than discrete knowledge, skills and attitudes. We believe this categorization accounts for the integrated nature of competencies, and allows us to better visualize the competencies relevant to the key processes critical to IKT.

#### Defining consensus for core competencies

Our a priori definition of a highly rated competency consisted of an overall median rating of ‘Very Important’ or ‘Extremely Important’ in addition to at least 70% of respondents agreeing with either of those ratings within a round. In combination, median score and percent agreement is the most commonly used criteria for consensus based on a systematic review of health care Delphi studies [24]. We considered there to be consensus for a core competency if that competency was highly rated in two rounds. Competencies that did not meet the highly rated criteria for two rounds were excluded from the final list of core competencies. Competencies that were rated twice and received inconsistent ratings between rounds (e.g., highly rated in Round 2 and not highly rated in Round 3) were resolved through group discussion by members of the research team (EY, SS and NMS).

#### Round 1

The aims of Round 1 were to (1) characterize the participants in terms of stakeholder group (KU or researcher) as well as work role and setting, (2) rate the importance of all initially identified competencies for KUs and researchers to engage in IKT, and (3) generate new competencies based on participants’ comments. The Round 1 questionnaire was reviewed by a member of the research team (NMS), and minor adjustments were made to improve content and clarity prior to administering the survey.

#### Round 2

The aims of Round 2 were to (1) provide feedback (median rating and percent of participants who rated the competency as either ‘Very Important’ or ‘Extremely Important’) on the competencies rated in Round 1, (2) re-rate the importance of all competencies from the initial list to assess for consistency between rounds, (3) rate the importance of newly added competencies, and (4) generate new competencies based on participants’ comments if they arise.

#### Round 3

The aims of Round 3 were to (1) provide feedback (median rating and percent of participants who rated the competency as either ‘Very Important’ or ‘Extremely Important’) on the competencies rated in Round 2 that were being rated again, (2) re-rate the importance of initial competencies that received inconsistent ratings between Round 1 and Round 2, and (3) re-rate the importance of competencies that were added after Round 1 based on participants’ comments.

### Data analysis

After each round, we calculated the overall median rating and the percent of the participants who rated each competency as ‘Very Important’ or ‘Extremely Important’ (percent agreement). We also conducted a *post-hoc* analysis of the median rating and percent agreement for the individual KU and researcher groups since there was an unplanned difference between the number of KUs and researchers in the sample. To determine whether one group influenced the overall ratings of competencies, we followed the steps outlined by Elwyn et al. [30] to calculate the equimedian for competencies where there was discordance between the median rating for the KU and researcher groups (i.e., one group median was “Very Important” or above, and the other group median was below “Very Important”). The equimedian is the median of a cumulative distribution function that gives equal weight to the two groups’ responses. We used Microsoft Excel 2016 to perform the data analysis.

## Results

### Participants

Sixty-three potential participants were invited to participate in this study, 26 of whom responded to the first round of the questionnaire, representing a 41.3% response rate (Table 1). One participant did not provide an email address and therefore could not be contacted in subsequent rounds. Twenty-two out of the 26 participants from round 1 completed the questionnaire for the second round (84.6% retention rate) and 18 of 22 participants responded to the third round of the survey, representing an 81.8% retention rate.

**Table 1:** Characteristics of participants (Rounds 1, 2, and 3)

	Round 1 (*n* = 26)	Round 2 (*n* = 22)	Round 3 (*n* = 18)
	No. of respondents (%)		
**IKT stakeholder Group**			
Knowledge user	7 (26.9)	7 (31.8)	5 (27.8)
Researcher	19 (73.1)	15 (68.2)	13 (72.2)
**Predominant type of work**			
Research	16 (61.5)	14 (63.6)	13 (72.2)
Clinical work (patient care)	2 (7.7)	1 (4.6)	1 (5.6)
Program evaluation	1 (3.9)	1 (4.6)	0 (0)
Education and training	1 (3.9)	0 (0)	0 (0)
Other (e.g., program development, research support, policy analysis)	6 (23.1)	6 (27.3)	4 (22.2)
**Predominant work setting**			
Research – academic institution	7 (26.9)	6 (27.3)	5 (27.8)
Research – hospital	5 (19.2)	5 (22.7)	5 (27.8)
Education – academic institution	5 (19.2)	3 (13.6)	3 (16.7)
Administration – government	2 (7.7)	2 (9.1)	1 (5.6)
Clinical – hospital	1 (3.9)	0 (0)	0 (0)
Administration – hospital	1 (3.9)	1 (4.5)	0 (0)
Other (e.g., community organizations, research foundations, professional associations)	5 (19.2)	5 (22.7)	4 (22.2)

Participants were asked to identify the predominant role that they fill in the IKT process, either KU or researcher. Researchers represented approximately 68 to 73% of the participants in each round (Table 1). In an open-ended comment section in Round 1, two participants reported that they consider themselves to fill the role of KU and researcher, although both participants selected researcher as their predominant role.

In round 1, 7 KUs and 19 researchers responded. The most common type of work was program development/evaluation (*n* = 2, 29%) and research support (*n* = 2, 29%) for KUs, with research (*n* = 16, 84%) being the most common type of work for researchers. KUs worked primarily in government (*n* = 2, 29%) and ‘Other’ settings (*n* = 4, 57%), and researchers primarily worked in academic institutions (*n* = 11, 58%) or hospital (*n* = 5, 26%) settings.

### Round 1

Of the 59 initial competencies (KU competencies =29, researcher competencies = 30), 43 (72.9%) met the highly rated criteria (KU competencies = 16, researcher competencies = 27). The majority of the KU competencies that did not meet the highly rated criteria (9/16, 56.3%) were from the ‘Evidence’ domain. Based on the participants’ comments, we identified seven new competencies (KU competencies = 5, researcher competencies = 2) to include in subsequent rounds (Table 2).

**Table 2:** New competencies identified by participants in Round 1

**Knowledge users** 1. Advocate for inclusion of appropriate knowledge users in the IKT process (evidence domain) 2. Understand the resource implications (e.g. funding, time) of the IKT process (evidence domain) 3. Implement actionable strategies to ensure all team members remain accountable for their expected contributions throughout the process (evidence domain) 4. Differentiate between evaluation, research, and quality improvement (evidence domain) 5. Utilize various resources (e.g., resource librarians) for evidence-gathering activities (evidence domain) 6. Clarify the roles of individual team members (teamwork domain)	
**Researcher** 7. Understand how intellectual property considerations may impact the dissemination process	

### Round 2

Of the 43 competencies that met the highly rated criteria from Round 1, nine (20.9%) (KU competencies = 2, researcher competencies = 7) fell below the threshold after Round 2 and were subsequently eliminated. All 16 (100%) competencies that did not meet the highly rated criteria in Round 1 also did not meet these criteria in Round 2, demonstrating consistency between rounds. One (14.3%) of the seven new competencies that were added after Round 1 was rated highly. Participants did not identify new competencies after Round 2.

### Round 3

The nine (100%) competencies that showed inconsistency between Round 1 and Round 2 (i.e., met the highly rated criteria in Round 1 but not in Round 2), were rated highly in Round 3, and were therefore retained in the final list of core competencies (Table 3). Of the 7 new competencies that were added in Round 2, one (14.3%) remained consistently highly rated, four (57.1%) remained consistently below the highly rated threshold, and two (28.6%) met the highly rated criteria in Round 3 despite missing the threshold in Round 2. Members of the research team discussed the two competencies that demonstrated inconsistency between Rounds 2 and 3 and resolved to include them in the final list of prioritized competencies for IKT.

**Table 3:** List of core competencies for knowledge users and researchers to engage in IKT after 3 survey rounds

	Competency	ROUND 1			ROUND 2			ROUND 3		
		Median (% agree)	Median (% agree)	Median (% Agree)	Median (% agree)	Median (% agree)	Median (% Agree)	Median (% agree)	Median (% agree)	Median (% Agree)
		KU	Researcher	Pooled	KU	Researcher	Pooled	KU	Researcher	Pooled
		*n* = 7 (unless otherwise indicated)	*n* = 19 (unless otherwise indicated)	*n* = 26 (unless otherwise indicated)	*n* = 7 (unless otherwise indicated)	*n* = 15 (unless otherwise indicated)	*n* = 22 (unless otherwise indicated)	*n* = 5 (unless otherwise indicated)	*n* = 13 (unless otherwise indicated)	*n* = 18 (unless otherwise indicated)
**Knowledge User**										
**Evidence Domain**										
1	Apply different types of knowledge to inform decision-making	Extremely Important (71.4)	Very Important (84.2)	Very Important (80.8)	Very Important (100)	Very Important (80.0)	Very Important (86.4)			
2	Identify decision-makers’ information needs and priorities	Very Important (85.7)	Very Important (84.2)	Very Important (84.6)	Very Important (100)	Very Important (93.3)	Very Important (95.5)			
3	Understand how local healthcare system factors (e.g. health services, health literacy) impact decision making processes	Extremely Important (71.4)	Very Important/ Extremely Important (94.4) *n = 18	Extremely Important (88.0) *n = 25	Very Important (57.1)	Very Important (73.3)	Very Important (68.2)	Very Important (100)	Very Important (100)	Very Important (100.0)
**Teamwork Domain**										
1	Build healthy working relationships with other team members	Extremely Important (100)	Extremely Important (94.7)	Extremely Important (96.2)	Very Important (85.7)	Extremely Important (86.7)	Extremely Important (86.4)			
2	Foster productive networks of researchers and decision makers	Important (28.6)	Extremely Important (94.7)	Very Important (76.9)	Very Important (85.7)	Very Important (73.3)	Very Important (77.3)			
3	Create opportunities to learn and share knowledge through informal and formal means	Extremely Important (71.4)	Extremely Important (94.7)	Extremely Important (88.5)	Very Important (85.7)	Very Important (66.7)	Very Important (72.7)			
4	Demonstrate and promote appropriate attitudes and behaviours when working with marginalized or vulnerable populations	Extremely Important (85.7)	Extremely Important (78.9)	Extremely Important (80.8)	Extremely Important (83.3) *n = 6	Extremely Important (80.0)	Extremely Important (81.0) *n = 21			
5	Value and contribute to knowledge sharing activities	Extremely Important (85.7)	Extremely Important (89.5)	Extremely Important (88.5)	Very Important (85.7)	Very Important (80.0)	Very Important (81.8)			
6	Advocate for inclusion of appropriate knowledge users in the IKT process				Very Important (71.4)	Very Important (73.3)	Very Important (72.7)	Very Important (100)	Very Important (100)	Very Important (100.0)
**KT Activities Domain**										
1	Address barriers and facilitators to applying knowledge to policy/decision-making	Very Important (71.4)	Very Important (89.5)	Very Important (84.6)	Very Important (85.7)	Very Important (80.0)	Very Important (81.8)			
2	Interact with knowledge brokers (an intermediary who links knowledge sources, and knowledge itself to organizations in its network) to assist with developing and/or finding and implementing evidence	Very Important (71.4)	Very Important (78.9)	Very Important (76.9)	Very Important (100)	Very Important (66.7)	Very Important (77.3)			
3	Identify practice gaps and opportunities to use relevant evidence to improve practice	Very Important (100)	Extremely Important (89.5)	Very Important/ Extremely Important (92.3)	Very Important (85.7)	Very Important (80.0)	Very Important (81.8)			
4	Identify and address inconsistencies between research findings and expertise or patient preferences	Extremely Important (85.7)	Very Important (84.2)	Very Important (84.6)	Very Important (71.4)	Very Important (60.0)	Very Important (63.6)	Very Important (100)	Very Important (92.3)	Very Important (94.4)
5	Describe how the patient’s values affect the balance between potential advantages and disadvantages of available healthcare/policy options	Very Important (85.7)	Very Important (84.2)	Very Important (84.6)	Very Important (85.7)	Very Important (73.3)	Very Important (77.3)			
6	Appropriately involve the patient in decision-making	Extremely Important (71.4)	Extremely Important (84.2)	Extremely Important (80.8)	Extremely Important (71.4)	Extremely Important (80.0)	Extremely Important (77.3)			
7	Promote the use of research and outcome data to formulate, evaluate and/or revise policy and practices to improve care	Extremely Important (100)	Extremely Important (89.5)	Extremely Important (92.3)	Extremely Important (100)	Very Important (100)	Very Important (100.0)			
8	Adapt and apply the evidence for the local practice context/environment	Extremely Important (85.7)	Extremely Important (100)	Extremely Important (96.2)	Extremely Important (85.7)	Very Important (86.7)	Very Important/ Extremely Important (86.4)			
9	Understand the resource implications (e.g. funding, time) of the IKT process				Very Important (57.1)	Very Important (53.3)	Very Important (54.5)	Very Important (80.0)	Very Important (69.2)	Very Important (72.2)
		**ROUND 1**			**ROUND 2**			**ROUND 3**		
		Median (% agree)	Median (% agree)	Median (% Agree)			Median (% Agree)	Median (% agree)	Median (% agree)	Median (% Agree)
		**KU**	**Researcher**	**Pooled**	**KU**	**Researcher**	**Pooled**	**KU**	**Researcher**	**Pooled**
		*n* = 7 (unless otherwise indicated)	*n* = 19 (unless otherwise indicated)	*n* = 26 (unless otherwise indicated)	*n* = 7 (unless otherwise indicated)	*n* = 15 (unless otherwise indicated)	*n* = 22 (unless otherwise indicated)	*n* = 5 (unless otherwise indicated)	*n* = 13 (unless otherwise indicated)	*n* = 18 (unless otherwise indicated)
**Researcher**										
**Evidence Domain**										
1	Understand how different types of knowledge (e.g. research, practice, theory) are generated and used in KT	Extremely Important (100)	Extremely Important (94.7)	Extremely Important (96.2)	Extremely Important (71.4)	Extremely Important (86.7)	Extremely Important (81.8)			
2	Apply appropriate research methodologies to examine the determinants of knowledge use across different settings and stakeholder groups	Extremely Important (100)	Extremely Important (94.7)	Extremely Important (96.2)	Extremely Important (100)	Extremely Important (86.7)	Extremely Important (90.9)			
3	Design and evaluate the impact, effectiveness and sustainability of KT strategies in different settings	Very Important (85.7)	Extremely Important (94.7)	Extremely Important (92.3)	Extremely Important (100)	Extremely Important (73.3)	Extremely Important (81.8)			
4	Respond to questions by stakeholders regarding the evidence generated to inform decision-making	Extremely Important (71.4)	Extremely Important (100)	Extremely Important (92.3)	Extremely Important (100)	Very Important (80.0)	Very Important (86.4)			
5	Apply the most appropriate dissemination tool for communicating with different audiences/stakeholders	Very Important (57.1)	Very Important (84.2)	Very Important (76.9)	Very Important (85.7)	Very Important (73.3)	Very Important (77.3)			
6	Incorporate the most relevant stakeholder perspectives into the research process and implementation cycle	Very Important (100)	Extremely Important (100)	Extremely Important (100.0)	Extremely Important (100)	Extremely Important (86.7)	Extremely Important (90.9)			
7	Select appropriate KT models or frameworks of knowledge dissemination and implementation for the context being considered	Very Important (85.7)	Extremely Important (83.3) *n = 18	Extremely Important (84.0) *n = 25	Very Important (85.7)	Extremely Important (73.3)	Very Important (77.3)			
8	Help transform clinical, management or policy questions into research questions	Very Important (85.7)	Extremely Important (94.7)	Very Important/ Extremely Important (92.3)	Very Important (85.7)	Very Important (80.0)	Very Important (81.8)			
**Teamwork Domain**										
1	Use effective communication strategies within the context being considered	Extremely Important (100)	Extremely Important (100)	Extremely Important (100.0)	Very Important (85.7)	Very Important (73.3)	Very Important (77.3)			
2	Use effective strategies to set priorities and manage/resolve conflict between stakeholders with differing interests	Important (42.9)	Very Important (84.2)	Very Important (73.1)	Very Important (71.4)	Very Important (60.0)	Very Important (63.6)	Very Important (80.0)	Very Important (69.2)	Very Important (72.2)
3	Evaluate the impact of knowledge brokering to connect evidence to practice/policy	Very Important (85.7)	Very Important (68.4)	Very Important (73.1)	Very Important (71.4)	Very Important (60.0)	Very Important (63.6)	Very Important (100)	Very Important (61.5)	Very Important (72.2)
4	Demonstrate and promote appropriate attitudes and behaviours when working with marginalized or vulnerable populations	Extremely Important (66.7) *n = 6	Extremely Important (89.5)	Extremely Important (84.0) *n = 25	Extremely Important (85.7)	Extremely Important (73.3)	Extremely Important (77.3)			
5	Form sustainable working relationships with relevant partners (e.g. government, industry, academia, funders etc)	Extremely Important (85.7)	Extremely Important (94.7)	Extremely Important (92.3)	Extremely Important (100)	Very Important (80.0)	Very Important/ Extremely Important (86.4)			
6	Advocate for appropriate change or action(s)	Very Important (57.1)	Extremely Important (78.9)	Very Important (73.1)	Very Important (57.1)	Very Important (60.0)	Very Important (59.1)	Very Important (80.0)	Very Important (76.9)	Very Important (77.8)
7	Form collaborative networks of relevant stakeholders to effectively generate, disseminate, and collate knowledge throughout the KT process	Very Important (100)	Extremely Important (89.5)	Very Important/ Extremely Important (92.3)	Very Important (85.7)	Very Important (66.7)	Very Important (72.7)			
8	Implement actionable strategies to ensure all team members remain accountable for their expected contributions throughout the process				Very Important (71.4)	Very Important (53.3)	Very Important (59.1)	Very Important (60.0)	Very Important (76.9)	Very Important (72.2)
**KT Activities Domain**										
1	Identify the most appropriate approach (es) to closing the knowledge-to-action gaps in the context being considered	Very Important (57.1)	Extremely Important (84.2)	Extremely Important (76.9)	Very Important (71.4)	Extremely Important (73.3)	Extremely Important (72.7)			
2	Develop and prioritize the steps in a dissemination plan within the research design	Very Important (71.4)	Very Important (89.5)	Very Important (84.6)	Very Important (71.4)	Very Important (66.7)	Very Important (68.2)	Very Important (100)	Very Important (100)	Very Important (100.0)
3	Consider the individual, organizational and system-level barriers and facilitators to knowledge uptake in planning KT activities	Very Important (71.4)	Extremely Important (94.7)	Extremely Important (88.5)	Very Important (71.4)	Extremely Important (86.7)	Extremely Important (81.8)			
4	Create KT plans that are closely linked to the goals of the research project	Very Important (57.1)	Very Important/ Extremely Important (94.4) *n = 18	Very Important (84.0) *n = 25	Very Important (100)	Extremely Important (80.0)	Very Important/ Extremely Important (86.4)			
5	Incorporate patient’s values into KT plan by balancing potential advantages and disadvantages of available options	Very Important (85.7)	Extremely Important (89.5)	Very Important (88.5)	Very Important (85.7)	Extremely Important (86.7)	Very Important (86.4)			
6	Conduct stakeholder analyses to understand the target audiences, interest in and capacity to engage with the evidence	Very Important (71.4)	Extremely Important (84.2)	Very Important/ Extremely Important (80.8)	Extremely Important (71.4)	Very Important (60.0)	Very Important (63.6)	Very Important (100)	Very Important (92.3)	Very Important (94.4)
7	Work collaboratively with decision/policy makers to synthesize and develop tailored messages for the target audience	Very Important (85.7)	Extremely Important (100)	Extremely Important (96.2)	Very Important (85.7)	Very Important (80.0)	Very Important (81.8)			
8	Create strategies to collect, collate and package evidence in an accessible and relevant manner for policy and practice	Very Important (71.4)	Extremely Important (94.7)	Very Important (88.5)	Very Important (71.4)	Very Important (73.3)	Very Important (72.7)			
9	Develop a systematic and inclusive KT plan that addresses the critical aspects of project implementation and management	Very Important (57.1)	Extremely Important (89.5)	Very Important (80.8)	Important (42.9)	Extremely Important (73.3)	Very Important (63.6)	Very Important (100)	Very Important (100)	Very Important (100.0)
10	Identify various roles of KT partners and practitioners in enhancing user engagement	Important (28.6)	Very Important (89.5)	Very Important (73.1)	Very Important (57.1)	Very Important (66.7)	Very Important (63.6)	Very Important (100)	Very Important (84.6)	Very Important (88.9)
11	Design KT strategies that include program-level and organizational-level KT	Very Important (57.1)	Extremely Important (89.5)	Very Important (80.8)	Very Important (71.4)	Very Important (73.3)	Very Important (72.7)			
12	Use tools to support knowledge production processes such as: ethics approval, collaboration agreements, and shared decision-making structures	Very Important (71.4)	Very Important (84.2)	Very Important (80.8)	Very Important (85.7)	Very Important/ Extremely Important (71.4) *n = 14	Very Important (76.2) *n = 21			

“% Agree” refers to the percentage of participants who agreed with either “Very Important” or “Extremely Important”

### Core competencies for IKT

A total of 46 competencies were identified as highly important for IKT in our study (Table 3). Eighteen competencies were deemed core competencies for KUs (Evidence = 3, Teamwork = 6, Knowledge Translation Activities = 9), and there were 28 core competencies for researchers (Evidence = 8, Teamwork = 8, Knowledge Translation Activities = 12).

### Competencies eliminated during the Delphi process

A total of 20 competencies from the initial list were eliminated (Table 4). Sixteen of these were KU competencies (Evidence = 11, Teamwork = 3, Knowledge Translation Activities = 2) and 4 of these were researcher competencies (Evidence = 1, Teamwork = 1, Knowledge Translation Activities = 2).

**Table 4:** List of eliminated competencies for knowledge users and researchers to engage in IKT after 3 survey rounds

	Competency	ROUND 1			ROUND 2			ROUND 3		
		Median (% agree)	Median (% agree)	Median (% agree)	Median (% agree)	Median (% agree)	Median (% agree)	Median (% agree)	Median (% agree)	Median (% agree)
		KU	Researcher	Pooled	KU	Researcher	Pooled	KU	Researcher	Pooled
		*n* = 7 (unless otherwise indicated)	*n* = 19 (unless otherwise indicated)	*n* = 26 (unless otherwise indicated)	*n* = 7 (unless otherwise indicated)	*n* = 15 (unless otherwise indicated)	*n* = 22 (unless otherwise indicated)	*n* = 5 (unless otherwise indicated)	*n* = 13 (unless otherwise indicated)	*n* = 18 (unless otherwise indicated)
**Knowledge User**										
**Evidence Domain**										
1	Critically appraise research findings	Very Important (71.4)	Important (31.6)	Important (42.3)	Very Important (57.1)	Important (6.7)	Important (22.7)			
2	Identify and select the most appropriate evidence for the context being considered	Very Important (57.1)	Very Important (68.4)	Very Important (65.4)	Very Important (85.7)	Important/ Very Important (50.0) *n = 14	Very Important (61.9) *n = 21			
3	Develop research questions for literature searches	Very Important (71.4)	Important (31.6)	Important (42.3)	Important (0.0)	Important (7.1) *n = 14	Important (4.8) *n = 21			
4	Create and execute an efficient search strategy within relevant electronic databases	Slightly Important (42.9)	Slightly Important (26.3)	Slightly Important (30.8)	Slightly Important (0.0)	Slightly Important (0.0) *n = 14	Slightly Important (0.0) *n = 21			
5	Keep up to date on relevant literature for the context being considered	Important (42.9)	Important (47.4)	Important (46.2)	Important (28.6)	Important (28.6) *n = 14	Important (28.6) *n = 21			
6	Explain the different types of knowledge (e.g. research, practice, theory) that contribute to decision-making	Important (42.9)	Important/ Very Important (50) *n = 18	Important (48) *n = 25	Important (0.0)	Important (14.3) *n = 14	Important (9.5) *n = 21			
7	Examine and interpret selected evidence from a literature search	Important (42.9)	Important (47.4)	Important (46.2)	Important (0.0)	Important (7.1) *n = 14	Important (4.8) *n = 21			
8	Use implementation resources (e.g. knowledge translation tools) to apply evidence for multiple audiences	Very Important (85.7)	Very Important (57.9)	Very Important (65.4)	Very Important (71.4)	Very Important (64.3) *n = 14	Very Important (66.7) *n = 21			
9	Describe the research process (e.g. research question, research ethics, different methodologies, data collection and analyses)	Important (14.3)	Important (31.6)	Important (26.9)	Important (14.3)	Slightly Important (0.0) *n = 13	Slightly Important (5.0) *n = 20			
10	Differentiate between evaluation, research, and quality improvement				Very Important (85.7)	Slightly Important/ Important (21.4) *n = 14	Important (42.9) *n = 21	Very Important (60.0)	Important (23.1)	Important (33.3)
11	Utilize various resources (e.g., resource librarians) for evidence-gathering activities				Important (42.9)	Slightly Important (0.0) *n = 14	Important (14.3) *n = 21	Important (40.0)	Slightly Important (7.7)	Slightly Important (16.7)
**Teamwork Domain**										
1	Help others access and appraise evidence	Important (28.6)	Important (42.1)	Important (38.5)	Important (14.3)	Slightly Important/ Important (0.0) *n = 14	Important (4.8) *n = 21			
2	Actively seek opportunities to engage in the research process	Important (42.9)	Important (47.4)	Important (46.2)	Important (14.3)	Slightly Important/ Important (0.0) *n = 14	Important (4.8) *n = 21			
3	Clarify the roles of individual team members				Very Important (57.1)	Important (26.7)	Important (36.4)	Very Important (60.0)	Important (33.3) *n = 12	Important (41.2) *n = 17
**KT Activities Domain**										
1	Utilize appropriate KT framework(s) for the context being considered	Very Important (71.4)	Important (47.4)	Very Important (53.8)	Very Important (71.4)	Important (30.8) *n = 13	Important (45.0) *n = 20			
2	Lead the team in synthesizing research evidence to improve healthcare services	Important (28.6)	Important (36.8)	Important (34.6)	Important (42.9)	Slightly Important (0.0) *n = 14	Slightly Important (14.3) *n = 21			
		**ROUND 1**			**ROUND 2**			**ROUND 3**		
		Median (% agree)	Median (% agree)	Median (% agree)	Median (% agree)	Median (% agree)	Median (% agree)	Median (% agree)	Median (% agree)	Median (% agree)
		**KU**	**Researcher**	**Pooled**	**KU**	**Researcher**	**Pooled**	**KU**	**Researcher**	**Pooled**
		*n* = 7 (unless otherwise indicated)	*n* = 19 (unless otherwise indicated)	*n* = 26 (unless otherwise indicated)	*n* = 7 (unless otherwise indicated)	*n* = 15 (unless otherwise indicated)	*n* = 22 (unless otherwise indicated)	*n* = 5 (unless otherwise indicated)	*n* = 13 (unless otherwise indicated)	*n* = 18 (unless otherwise indicated)
**Researcher**										
**Evidence Domain**										
1	Identify and set priorities regarding policy needs and research options	Important (28.6)	Very Important (73.7)	Very Important (61.5)	Very Important (71.4)	Important/ Very Important (50.0) *n = 14	Very Important (57.1) *n = 21			
**Teamwork Domain**										
1	Understand how intellectual property considerations may impact the dissemination process				Very Important (71.4)	Important (46.7)	Very Important (54.5)	Very Important (80.0)	Very Important (61.5)	Very Important (66.7)
**KT Activities Domain**										
1	Articulate the difference between research and evaluation for a particular context	Very Important (71.4)	Very Important (63.2)	Very Important (65.4)	Very Important (71.4)	Important/ Very Important (50.0) *n = 14	Very Important (57.1) *n = 21			
2	Understand the role and practices of social media in the KT process	Important (42.9)	Very Important (63.2)	Very Important (57.7)	Very Important (85.7)	Important (42.9) *n = 14	Very Important (57.1) *n = 21			

“% Agree” refers to the percentage of participants who agreed with either “Very Important” or “Extremely Important”

### Discordance between KU and researcher groups

To determine if the unequal number of KUs and researchers had an impact on the outcome of the Delphi study, we conducted a post-hoc analysis for competencies that received discordant ratings between KU and researcher groups by calculating the equimedian, or weighted median, for each competency rating [30]. Equimedian values are presented in Supplemental Table 1 (core competencies) and Supplemental Table 2 (eliminated competencies). Based on this analysis, we concluded that use of the equimedian rather than the overall median as an a priori criterion for a high rating, would have resulted in very few (3%) of the competencies receiving a higher median rating. Therefore, the group influence on the overall ratings of the competencies was very small. Additionally, because a high rating was also dependent on the percent agreement with ‘Very Important’ or ‘Extremely Important’, using the equimedian would not have changed the outcomes of this study.

## Discussion

This study provides important insights regarding KU and researcher competencies that are perceived by study participants as important to engaging in IKT. Both KU and researcher participants agreed on a total of 46 competencies, with 18 competencies deemed important for KUs in the domains of Evidence (*n* = 3); Teamwork (*n* = 6), and Knowledge Translation Activities (*n* = 9); and 28 competencies rated as important for researchers under the same domains (Evidence (*n* = 8), Teamwork (*n* = 8), and Knowledge Translation Activities (*n* = 12)).

### ‘Evidence’ domain

The competencies prioritized for KUs and researchers in the ‘Evidence’ domain were complementary and support the sentiment that the partnerships formed between KUs and researchers are greater than the sum of its parts in the IKT context [31, 32].

#### Knowledge user competencies

The competencies from the ‘Evidence’ domain that were rated as highly valuable skills for KUs pertain to having a working familiarity with the local health care system and the use of different forms of knowledge in decision-making. This is consistent with assertions that a deep understanding of the context for KT activities and an appreciation for a range of evidence resources are critical for successful knowledge translation [18]. This is also in keeping with the conceptualization of evidence-based practice in which the application of different types of knowledge, including tacit knowledge, has been previously recognized as central to decision-making within a range of health care contexts [31, 33–35]. For IKT in particular, KUs are particularly well-positioned to bring unique context-related knowledge and perspectives to the development of research priorities, and the co-production and application of research evidence [31, 36].

Competencies in the ‘Evidence’ domain that were not prioritized for KUs included the ability to critically appraise research findings, create and execute an efficient search strategy within relevant electronic databases, examine and interpret selected evidence from a literature search, and keep up to date on relevant literature. This was a notable yet surprising finding given the importance attributed to developing research knowledge and skills for knowledge users such as health care professionals in the context of evidence-based practice [37]. Due to the low number of KUs in this study and their diverse roles, some participants may not have perceived these competencies as important for their day-to-day work, and competency requirements may vary across contexts. For example, while KUs serving in clinical roles may be expected to learn continuously about new clinical approaches, the same expectations may not be applied to KUs working in managerial roles with respect to the explicit use of research evidence. Issues of feasibility may also prevent all stakeholders from engaging with the entire IKT process in every circumstance [18]. Moreover, the competencies not prioritized for KUs may have been perceived as more relevant to the researcher role; suggesting that the knowledge-to-action gap may still be perceived as a *knowledge transfer* issue [36] rather than one of *knowledge co-production* involving KUs and researchers [38, 39]. Greater attention will need to be paid to interventions and curricula that facilitate attitudinal shifts toward beliefs and values that support the entire IKT process rather than simply focusing on knowledge transfer between distinct groups of researchers and knowledge users [18, 37].

#### Researcher competencies

The competencies in the ‘Evidence’ domain that were prioritized for researchers relate directly to the research process. This is not surprising since researchers are generally academic researchers who are directly involved in and skilled at carrying out the research process [32, 39]. Notably, “Identify and set priorities regarding policy needs and research options” was not a highly rated competency for researchers. In keeping with IKT theory and practice [17, 32], this may be because this competency was considered by study participants to be a role of the KUs who may be better suited to set priorities given their deep understanding of the context.

### ‘Teamwork’ domain

Our study findings elaborate on the competencies suggested by Mallidou et al. [18] who described collaborative skills in managing and leading teams as central to knowledge translation. Competencies within the ‘teamwork’ domain in our study were not only highly rated in all 3 Delphi rounds in this study, but were considered important for both KUs and researchers. They pertain to navigating the ‘boundary organizations’ occurring at the intersection between science and practice [13] and promoting ongoing partnerships between KUs and researchers. Consistent with other participatory knowledge co-production approaches, many of the highly rated ‘Teamwork’ competencies support KU and researchers’ roles in knowledge sharing and in forming and maintaining healthy relationships between KUs and researchers [14, 15, 40–42]. The knowledge co-production process is particularly vulnerable to the impact of power relationships amongst researchers and KUs, especially for issues of sustainability, and the integration of diverse perspectives and ideas [12]. Data from the present study support the development of specific ‘teamwork’ competencies to address these shortcomings and to minimize these common vulnerabilities, particularly when marginalized populations are involved. Among these are competencies related to the formation and maintenance of healthy relationships and networks throughout the research cycle to optimize team functioning; these include recognizing and valuing the expertise of all team members, and for KUs to advocate for their involvement in the IKT process. These competencies are well-aligned with community-based participatory approaches in which efforts aimed at fostering the team’s capacity for shared ownership of research results afforded opportunities for all team members to engage in meaningful dialogue [43]. However, the full potential of ‘teamwork’ competencies may only be optimally realized if the underlying values and commitment of team members are sufficiently oriented toward supporting IKT [18].

### ‘Knowledge translation activities’ domain

#### Knowledge user competencies

The KU competencies prioritized under the ‘KT Activities’ domain support activities typically executed towards the end of the research process that promote the application of research evidence to the local context. Notably, the two KU competencies that were eliminated from the ‘KT Activities’ domain were 1) utilizing appropriate KT framework(s) for the context being considered and 2) leading the team in synthesizing research evidence to improve healthcare services. Until more recently, both of these competencies have been traditionally associated with the researcher role [3]. Owing to their familiarity with stakeholder needs and the local environment in which knowledge is to be applied, some have argued for KUs, and specifically managers, to be at the helm for planning and executing KT interventions [38, 44]. Indeed, KUs may not view themselves as agents of change in a formal manner and may not recognize the implicit ways in which they are already applying KT frameworks in policy and practice. However, there is growing evidence to suggest that KUs can be effective leaders and change agents for implementing innovations, particularly in contexts where KUs are highly committed and proactively forming working relationships with community partners [45]. Thus, while team leadership in synthesizing research evidence was not prioritized as a KU competency in this study, it may still be worthwhile to examine the need to build capacity among individual KUs as well as organizations, noting specific conditions under which KUs may best serve as change agents and leaders over knowledge translation activities [17].

#### Researcher competencies

There is emerging evidence to suggest that there are potential benefits to blurring the lines between research, quality improvement (QI) and evaluation which has important implications for partnership formation between academic and health organizations [36]. Thus, it is surprising that recognizing the difference between research and evaluation in a particular context was not prioritized as a researcher competency. This is perhaps due to a lack of understanding of the potential opportunities afforded by overlapping research with QI and evaluation efforts. When research, QI and evaluation are conceptualized as distinct activities within an organization, it can inadvertently lead to an artificial separation of roles and responsibilities to the detriment of the IKT process. Thus, future work is warranted to explore how traditional conceptions of research could be reimagined [36] and the competencies required of KUs and researchers for this reconceptualization.

### Recommendations

Our study highlights the criticality of teamwork competencies to enable KUs and researchers from both sides of the ‘boundary organizations’ to interact effectively and efficiently to co-produce knowledge [12]. In the face of long-standing hierarchical traditions and power differentials within the research context, one possible way forward is to build team members’ capacity to create and/or contribute to research environments in which teamwork competencies and practices are promoted, normalized and made explicit. Creating a safe space for exchange of dialogue by setting ground rules, ensuring participant interactivity, and encouraging shared ownership of study results are examples of strategies to assist in managing power relations [43]. Central to these strategies is KUs and researchers’ capacity to be reflexive about one’s own values and assumptions, as well as the ability to communicate clearly and effectively [43, 46]. Notably, competency in reflexivity was not included in the initial Delphi survey, nor did study participants recommend this as a new competency to include in subsequent rounds. Thus, a potential focus of competency development to support IKT could be to build reflexive skills among KUs and researchers.

Evidence is also accumulating from studies of health care teams to suggest that training efforts should additionally attend to the collective competence of the entire team or of the organization [18, 46, 47]. Building individual competence may be necessary but insufficient to address the multitude of contextual factors facing each unique IKT scenario [46, 47]. Similarly, teams working in the IKT context must simultaneously address the expertise relevant to the task at hand as well as the processes unique to that context that allow for optimal collective functioning. Thus, further research is warranted to determine if and to what extent the notion of collective competence plays a role in the IKT context, and how team-based learning approaches may benefit the development of expertise in IKT.

Since a key ingredient for successful IKT is establishing a shared understanding of language, roles and responsibilities, and ultimately how decisions are made, it stands to reason that KUs and researchers should also be equipped to negotiate the IKT process. While negotiation was not an explicit competency in this study, we believe it is implicit in the KU competencies prioritized in this study, such as to ‘address inconsistencies between research findings and expert or patient preferences’, and ‘advocate for inclusion of appropriate KUs in the IKT process’. Similarly, strong negotiation skills are implicit in researcher competencies including setting research priorities and identifying the roles of partners in enhancing user engagement. Moreover, negotiation of roles and responsibilities to enable successful teamwork is important across diverse organizational and non-organizational contexts, as well as teams that represent multiple stakeholders. In a study of how nurse practitioners were integrated into primary health care teams, role negotiation and clarification was a critical determinant in the performance of health care teams [48]. In this case, role clarification was most effective when individuals mobilized their negotiation skills within the constraints of their respective organizational processes. Therefore, it may be prudent to draw on organizational development and transdisciplinary literatures, and health care team practices to identify ideal approaches to build capacity among KUs and researchers to negotiate effectively.

This study led to the exclusion of ‘leadership’ over KT activities as a KU competency in despite of literature pointing to the important role of KUs, such as managers, in implementation leadership [44, 45]. This could be reflective of persistent traditional paradigms of research in which leadership over KT activities is held by researchers. While the rewards of more collaborative models of leadership have been purported in the health care practice context [46, 47], wider adoption of similar models in IKT must be considered. As we strive to understand ways to improve implementation processes, a way forward may be to explore the extent to which domains of the Implementation Leadership Scale (proactive, knowledgeable, supportive and perseverant leadership) may be relevant to and fostered amongst KUs [49, 50]. Pursuing this line of research can potentially yield opportunities to invest in alternative means for KUs to contribute meaningfully to the IKT process.

### Limitations

We acknowledge that of the use of previously identified general KT competencies may have inadvertently de-emphasized or excluded competencies relevant to IKT, including those that underpin social processes important to IKT. Hence, future work could explore specific competencies, such as negotiation skills, that are critical to managing the evolving roles of KU and researcher throughout the research cycle. We also recognize that while some competencies did not meet the criteria in this study, they may still hold relevance for researchers and knowledge users in some contexts, and hence should not be ignored altogether. While we sought appropriate representation from researchers and knowledge user groups, participants were recruited from a single international IKT network that is dominated by Canadian knowledge users who might share a particular worldview about IKT. We also acknowledge the smaller number of KU participants in this study and the absence of data regarding gender identities. Future studies could examine the external validity of the competencies prioritized in this study for a wide range of research contexts, research partnerships and research co-production team roles assumed by KUs. This should include establishing competencies to support the formation of patient partners for projects that involve patient oriented research.

## Conclusion

Development of highly skilled KUs and researchers is critical if IKT teams are to effectively and efficiently co-produce research to address gaps in health care policy and practice. While core competencies have previously been identified to facilitate training in knowledge translation more generally [15], this study begins to clarify the competencies that are most relevant to KUs and researchers to support successful IKT. The results from this Delphi study specify competencies in three domains (‘evidence’, ‘knowledge ‘teamwork’ and ‘knowledge translation activities’) that are of particular importance for KUs and researchers to attend to when engaging in IKT. The competencies identified reflect the dynamic and complex nature of IKT wherein both KUs and researchers must be skilled in managing team dynamics and functions throughout all phases of the research process. Although competencies have been specified separately for KU and researchers in this study, future work could focus on how these can be further negotiated and contextualized due to the wide variations in IKT context, project and team

## Supplementary Information

**Additional file 1:**

## Data Availability

The datasets used and/or analysed during the current study are available from the corresponding author on reasonable request.

## Abbreviations

IKT: Integrated knowledge translation
IKTRN: Integrated Knowledge Translation Research Network
KTP: Knowledge Translation Pathway
KT: Knowledge translation
KU: Knowledge user
