# Supplementary material for: Identifying competencies for integrated knowledge translation: a Delphi study
Source: BMC Health Serv Res. 2021 Oct 30;21:1181. doi: 10.1186/s12913-021-07107-7 (PMC8556977; doi:10.1186/s12913-021-07107-7)
Supplement: Supplementary file 1 — Additional file 1: [file 12913_2021_7107_MOESM1_ESM.docx]

**SUPPLEMENTAL MATERIAL**

**Supplemental Table 1. List of core competencies for knowledge users and researchers to engage in IKT after 3 survey rounds.**

|  | **Competency** | **ROUND 1** | | | |  | **ROUND 2** | | | |  | **ROUND 3** | | | |
| --- | --- | --- | --- | --- | --- | --- | --- | --- | --- | --- | --- | --- | --- | --- | --- |
|  |  | Median  (% agree) | Median  (% agree) | Median  (% Agree) |  |  | Median  (% agree) | Median  (% agree) | Median  (% Agree) |  |  | Median  (% agree) | Median  (% agree) | Median  (% Agree) |  |
|  |  | **KU** | **Researcher** | **Pooled** | **Equi-median** |  | **KU** | **Researcher** | **Pooled** | **Equi-median** |  | **KU** | **Researcher** | **Pooled** | **Equi-median** |
|  |  | n=7 (unless otherwise indicated) | n=19  (unless otherwise indicated) | n=26 (unless otherwise indicated) |  |  | n=7 (unless otherwise indicated) | n=15 (unless otherwise indicated) | n=22 (unless otherwise indicated) |  |  | n=5 (unless otherwise indicated) | n=13 (unless otherwise indicated) | n=18 (unless otherwise indicated) |  |
|  | **Knowledge User** |  |  |  |  |  |  |  |  |  |  |  |  |  |  |
|  | **Evidence Domain** |  |  |  |  |  |  |  |  |  |  |  |  |  |  |
| 1 | Apply different types of knowledge to inform decision-making | Extremely Important (71.4) | Very Important  (84.2) | Very Important  (80.8) |  |  | Very Important  (100) | Very Important (80.0) | Very Important  (86.4) |  |  |  |  |  |  |
| 2 | Identify decision-makers’ information needs and priorities | Very Important (85.7) | Very Important (84.2) | Very Important  (84.6) |  |  | Very Important  (100) | Very Important (93.3) | Very Important  (95.5) |  |  |  |  |  |  |
| 3 | Understand how local healthcare system factors (e.g. health services, health literacy) impact decision making processes | Extremely Important (71.4) | Very Important/ Extremely Important  (94.4)  *n=18 | Extremely Important  (88.0)  *n=25 |  |  | Very Important  (57.1) | Very Important (73.3) | Very Important  (68.2) |  |  | Very Important (100) | Very Important (100) | Very Important  (100.0) |  |
|  | **Teamwork Domain** |  |  |  |  |  |  |  |  |  |  |  |  |  |  |
| 1 | Build healthy working relationships with other team members | Extremely Important  (100) | Extremely Important  (94.7) | Extremely Important  (96.2) |  |  | Very Important  (85.7) | Extremely Important (86.7) | Extremely Important  (86.4) |  |  |  |  |  |  |
| 2 | Foster productive networks of researchers and decision makers | *Important*  *(28.6)* | *Extremely Important*  *(94.7)* | *Very Important*  *(76.9)* | ***Very Important*** |  | Very Important  (85.7) | Very Important (73.3) | Very Important  (77.3) |  |  |  |  |  |  |
| 3 | Create opportunities to learn and share knowledge through informal and formal means | Extremely Important  (71.4) | Extremely Important  (94.7) | Extremely Important  (88.5) |  |  | Very Important  (85.7) | Very Important (66.7) | Very Important  (72.7) |  |  |  |  |  |  |
| 4 | Demonstrate and promote appropriate attitudes and behaviours when working with marginalized or vulnerable populations | Extremely Important  (85.7) | Extremely Important  (78.9) | Extremely Important  (80.8) |  |  | Extremely Important (83.3)  *n=6 | Extremely Important (80.0) | Extremely Important  (81.0)  *n=21 |  |  |  |  |  |  |
| 5 | Value and contribute to knowledge sharing activities | Extremely Important  (85.7) | Extremely Important  (89.5) | Extremely Important  (88.5) |  |  | Very Important  (85.7) | Very Important (80.0) | Very Important  (81.8) |  |  |  |  |  |  |
| 6 | Advocate for inclusion of appropriate knowledge users in the IKT process |  |  |  |  |  | Very Important (71.4) | Very Important (73.3) | Very Important  (72.7) |  |  | Very Important  (100) | Very Important  (100) | Very Important  (100.0) |  |
|  | **KT Activities Domain** |  |  |  |  |  |  |  |  |  |  |  |  |  |  |
| 1 | Address barriers and facilitators to applying knowledge to policy/decision-making | Very Important  (71.4) | Very Important  (89.5) | Very Important  (84.6) |  |  | Very Important  (85.7) | Very Important (80.0) | Very Important  (81.8) |  |  |  |  |  |  |
| 2 | Interact with knowledge brokers (an intermediary who links knowledge sources, and knowledge itself to organizations in its network) to assist with developing and/or finding and implementing evidence | Very Important  (71.4) | Very Important  (78.9) | Very Important  (76.9) |  |  | Very Important  (100) | Very Important (66.7) | Very Important  (77.3) |  |  |  |  |  |  |
| 3 | Identify practice gaps and opportunities to use relevant evidence to improve practice | Very Important  (100) | Extremely Important  (89.5) | Very Important/ Extremely Important  (92.3) |  |  | Very Important  (85.7) | Very Important (80.0) | Very Important  (81.8) |  |  |  |  |  |  |
| 4 | Identify and address inconsistencies between research findings and expertise or patient preferences | Extremely Important  (85.7) | Very Important  (84.2) | Very Important  (84.6) |  |  | Very Important  (71.4) | Very Important (60.0) | Very Important  (63.6) |  |  | Very Important  (100) | Very Important  (92.3) | Very Important  (94.4) |  |
| 5 | Describe how the patient’s values affect the balance between potential advantages and disadvantages of available healthcare/policy options | Very Important  (85.7) | Very Important  (84.2) | Very Important  (84.6) |  |  | Very Important  (85.7) | Very Important (73.3) | Very Important  (77.3) |  |  |  |  |  |  |
| 6 | Appropriately involve the patient in decision-making | Extremely Important  (71.4) | Extremely Important  (84.2) | Extremely Important  (80.8) |  |  | Extremely Important (71.4) | Extremely Important (80.0) | Extremely Important (77.3) |  |  |  |  |  |  |
| 7 | Promote the use of research and outcome data to formulate, evaluate and/or revise policy and practices to improve care | Extremely Important  (100) | Extremely Important  (89.5) | Extremely Important  (92.3) |  |  | Extremely Important (100) | Very Important (100) | Very Important  (100.0) |  |  |  |  |  |  |
| 8 | Adapt and apply the evidence for the local practice context/environment | Extremely Important  (85.7) | Extremely Important  (100) | Extremely Important  (96.2) |  |  | Extremely Important (85.7) | Very Important (86.7) | Very Important/ Extremely Important  (86.4) |  |  |  |  |  |  |
| 9 | Understand the resource implications (e.g. funding, time) of the IKT process |  |  |  |  |  | Very Important (57.1) | Very Important (53.3) | **Very Important**  **(54.5)** |  |  | Very Important  (80.0) | Very Important  (69.2) | Very Important  (72.2) |  |
|  |  |  |  |  |  |  |  |  |  |  |  |  |  |  |  |
|  |  | **ROUND 1** | | | |  | **ROUND 2** | | | |  | **ROUND 3** | | | |
|  |  | Median  (% agree) | Median  (% agree) | Median  (% Agree) |  |  |  |  | Median  (% Agree) |  |  | Median  (% agree) | Median  (% agree) | Median  (% Agree) |  |
|  |  | **KU** | **Researcher** | **Pooled** | **Equi-median** |  | **KU** | **Researcher** | **Pooled** | **Equi-median** |  | **KU** | **Researcher** | **Pooled** | **Equi-median** |
|  |  | n=7 (unless otherwise indicated) | n=19  (unless otherwise indicated) | n=26 (unless otherwise indicated) |  |  | n=7 (unless otherwise indicated) | n=15 (unless otherwise indicated) | n=22 (unless otherwise indicated) |  |  | n=5 (unless otherwise indicated) | n=13 (unless otherwise indicated) | n=18 (unless otherwise indicated) |  |
|  | **Researcher** |  |  |  |  |  |  |  |  |  |  |  |  |  |  |
|  | **Evidence Domain** |  |  |  |  |  |  |  |  |  |  |  |  |  |  |
| 1 | Understand how different types of knowledge (e.g. research, practice, theory) are generated and used in KT | Extremely Important  (100) | Extremely Important  (94.7) | Extremely Important  (96.2) |  |  | Extremely Important (71.4) | Extremely Important (86.7) | Extremely Important  (81.8) |  |  |  |  |  |  |
| 2 | Apply appropriate research methodologies to examine the determinants of knowledge use across different settings and stakeholder groups | Extremely Important  (100) | Extremely Important  (94.7) | Extremely Important  (96.2) |  |  | Extremely Important (100) | Extremely Important (86.7) | Extremely Important  (90.9) |  |  |  |  |  |  |
| 3 | Design and evaluate the impact, effectiveness and sustainability of KT strategies in different settings | Very Important  (85.7) | Extremely Important  (94.7) | Extremely Important  (92.3) |  |  | Extremely Important (100) | Extremely Important (73.3) | Extremely Important  (81.8) |  |  |  |  |  |  |
| 4 | Respond to questions by stakeholders regarding the evidence generated to inform decision-making | Extremely Important  (71.4) | Extremely Important  (100) | Extremely Important  (92.3) |  |  | Extremely Important (100) | Very Important (80.0) | Very Important  (86.4) |  |  |  |  |  |  |
| 5 | Apply the most appropriate dissemination tool for communicating with different audiences/stakeholders | Very Important  (57.1) | Very Important  (84.2) | Very Important  (76.9) |  |  | Very Important (85.7) | Very Important (73.3) | Very Important  (77.3) |  |  |  |  |  |  |
| 6 | Incorporate the most relevant stakeholder perspectives into the research process and implementation cycle | Very Important  (100) | Extremely Important  (100) | Extremely Important  (100.0) |  |  | Extremely Important (100) | Extremely Important (86.7) | Extremely Important  (90.9) |  |  |  |  |  |  |
| 7 | Select appropriate KT models or frameworks of knowledge dissemination and implementation for the context being considered | Very Important  (85.7) | Extremely Important  (83.3)  *n=18 | Extremely Important  (84.0)  *n=25 |  |  | Very Important (85.7) | Extremely Important (73.3) | Very Important  (77.3) |  |  |  |  |  |  |
| 8 | Help transform clinical, management or policy questions into research questions | Very Important  (85.7) | Extremely Important  (94.7) | Very Important/ Extremely Important  (92.3) |  |  | Very Important (85.7) | Very Important (80.0) | Very Important  (81.8) |  |  |  |  |  |  |
|  | **Teamwork Domain** |  |  |  |  |  |  |  |  |  |  |  |  |  |  |
| 1 | Use effective communication strategies within the context being considered | Extremely Important  (100) | Extremely Important  (100) | Extremely Important  (100.0) |  |  | Very Important (85.7) | Very Important (73.3) | Very Important  (77.3) |  |  |  |  |  |  |
| 2 | Use effective strategies to set priorities and manage/resolve conflict between stakeholders with differing interests | *Important*  *(42.9)* | *Very Important*  *(84.2)* | *Very Important*  *(73.1)* | ***Very Important*** |  | Very Important (71.4) | Very Important (60.0) | **Very Important**  **(63.6)** |  |  | Very Important  (80.0) | Very Important  (69.2) | Very Important  (72.2) |  |
| 3 | Evaluate the impact of knowledge brokering to connect evidence to practice/policy | Very Important  (85.7) | Very Important  (68.4) | Very Important  (73.1) |  |  | Very Important (71.4) | Very Important (60.0) | **Very Important**  **(63.6)** |  |  | Very Important  (100) | Very Important  (61.5) | Very Important  (72.2) |  |
| 4 | Demonstrate and promote appropriate attitudes and behaviours when working with marginalized or vulnerable populations | Extremely Important  (66.7)  *n=6 | Extremely Important  (89.5) | Extremely Important  (84.0)  *n=25 |  |  | Extremely Important  (85.7) | Extremely Important (73.3) | Extremely Important  (77.3) |  |  |  |  |  |  |
| 5 | Form sustainable working relationships with relevant partners (e.g. government, industry, academia, funders etc) | Extremely Important  (85.7) | Extremely Important  (94.7) | Extremely Important  (92.3) |  |  | Extremely Important  (100) | Very Important (80.0) | Very Important/ Extremely Important  (86.4) |  |  |  |  |  |  |
| 6 | Advocate for appropriate change or action(s) | Very Important  (57.1) | Extremely Important  (78.9) | Very Important  (73.1) |  |  | Very Important (57.1) | Very Important (60.0) | **Very Important**  **(59.1)** |  |  | Very Important  (80.0) | Very Important  (76.9) | Very Important  (77.8) |  |
| 7 | Form collaborative networks of relevant stakeholders to effectively generate, disseminate, and collate knowledge throughout the KT process | Very Important  (100) | Extremely Important  (89.5) | Very Important/ Extremely Important  (92.3) |  |  | Very Important  (85.7) | Very Important (66.7) | Very Important  (72.7) |  |  |  |  |  |  |
| 8 | Implement actionable strategies to ensure all team members remain accountable for their expected contributions throughout the process |  |  |  |  |  | Very Important (71.4) | Very Important (53.3) | **Very Important**  **(59.1)** |  |  | Very Important  (60.0) | Very Important  (76.9) | Very Important  (72.2) |  |
|  | **KT Activities Domain** |  |  |  |  |  |  |  |  |  |  |  |  |  |  |
| 1 | Identify the most appropriate approach(es) to closing the knowledge-to-action gaps in the context being considered | Very Important  (57.1) | Extremely Important  (84.2) | Extremely Important  (76.9) |  |  | Very Important (71.4) | Extremely Important  (73.3) | Extremely Important  (72.7) |  |  |  |  |  |  |
| 2 | Develop and prioritize the steps in a dissemination plan within the research design | Very Important  (71.4) | Very Important  (89.5) | Very Important  (84.6) |  |  | Very Important (71.4) | Very Important (66.7) | **Very Important**  **(68.2)** |  |  | Very Important  (100) | Very Important  (100) | Very Important  (100.0) |  |
| 3 | Consider the individual, organizational and system-level barriers and facilitators to knowledge uptake in planning KT activities | Very Important  (71.4) | Extremely Important  (94.7) | Extremely Important  (88.5) |  |  | Very Important (71.4) | Extremely Important  (86.7) | Extremely Important  (81.8) |  |  |  |  |  |  |
| 4 | Create KT plans that are closely linked to the goals of the research project | Very Important  (57.1) | Very Important/ Extremely Important  (94.4)  *n=18 | Very Important  (84.0)  *n=25 |  |  | Very Important (100) | Extremely Important  (80.0) | Very Important/ Extremely Important  (86.4) |  |  |  |  |  |  |
| 5 | Incorporate patient's values into KT plan by balancing potential advantages and disadvantages of available options | Very Important  (85.7) | Extremely Important  (89.5) | Very Important  (88.5) |  |  | Very Important (85.7) | Extremely Important  (86.7) | Very Important  (86.4) |  |  |  |  |  |  |
| 6 | Conduct stakeholder analyses to understand the target audiences, interest in and capacity to engage with the evidence | Very Important  (71.4) | Extremely Important  (84.2) | Very Important/ Extremely Important  (80.8) |  |  | Extremely Important (71.4) | Very Important (60.0) | **Very Important**  **(63.6)** |  |  | Very Important  (100) | Very Important  (92.3) | Very Important  (94.4) |  |
| 7 | Work collaboratively with decision/policy makers to synthesize and develop tailored messages for the target audience | Very Important  (85.7) | Extremely Important  (100) | Extremely Important  (96.2) |  |  | Very Important (85.7) | Very Important (80.0) | Very Important  (81.8) |  |  |  |  |  |  |
| 8 | Create strategies to collect, collate and package evidence in an accessible and relevant manner for policy and practice | Very Important  (71.4) | Extremely Important  (94.7) | Very Important  (88.5) |  |  | Very Important (71.4) | Very Important (73.3) | Very Important (72.7) |  |  |  |  |  |  |
| 9 | Develop a systematic and inclusive KT plan that addresses the critical aspects of project implementation and management | Very Important  (57.1) | Extremely Important  (89.5) | Very Important  (80.8) |  |  | *Important (42.9)* | *Extremely Important (73.3)* | **Very Important**  **(63.6)** | ***Very Important*** |  | Very Important  (100) | Very Important  (100) | Very Important  (100.0) |  |
| 10 | Identify various roles of KT partners and practitioners in enhancing user engagement | *Important*  *(28.6)* | *Very Important*  *(89.5)* | *Very Important*  *(73.1)* | ***Very Important*** |  | Very Important (57.1) | Very Important (66.7) | **Very Important**  **(63.6)** |  |  | Very Important  (100) | Very Important  (84.6) | Very Important  (88.9) |  |
| 11 | Design KT strategies that include program-level and organizational-level KT | Very Important  (57.1) | Extremely Important  (89.5) | Very Important  (80.8) |  |  | Very Important (71.4) | Very Important (73.3) | Very Important  (72.7) |  |  |  |  |  |  |
| 12 | Use tools to support knowledge production processes such as: ethics approval, collaboration agreements, and shared decision-making structures | Very Important  (71.4) | Very Important  (84.2) | Very Important  (80.8) |  |  | Very Important (85.7) | Very Important/ Extremely Important (71.4)  *n=14 | Very Important  (76.2)  *n=21 |  |  |  |  |  |  |

“% Agree” refers to the percentage of participants who agreed with either “Very Important” or “Extremely Important.”

Italicized text: indicate instances where there was discordance between KU and researcher group median ratings (i.e., for one group median was “Very Important” or above, and for the other group the median was below “Very Important”)

Bolded and Italicized text: signify that the equimedian rating is concordant with the pooled median rating for that competency

Bolded and capitalized text: signify that the equimedian rating is discordant with the pooled median rating for that competency

Bolded and underlined ratings: represent pooled median ratings that did not meet the highly rated criteria

**Supplemental Table 2. List of eliminated competencies for knowledge users and researchers to engage in IKT after 3 survey rounds.**

|  | **Competency** | **ROUND 1** |  |  |  |  | **ROUND 2** |  |  |  |  | **ROUND 3** |  |  |  |
| --- | --- | --- | --- | --- | --- | --- | --- | --- | --- | --- | --- | --- | --- | --- | --- |
|  |  | Median  (% agree) | Median  (% agree) | Median  (% agree) |  |  | Median  (% agree) | Median  (% agree) | Median  (% agree) |  |  | Median  (% agree) | Median  (% agree) | Median  (% agree) |  |
|  |  | **KU** | **Researcher** | **Pooled** | **Equi-median** |  | **KU** | **Researcher** | **Pooled** | **Equi-median** |  | **KU** | **Researcher** | **Pooled** | **Equi-median** |
|  |  | n=7 (unless otherwise indicated) | n=19  (unless otherwise indicated) | n=26 (unless otherwise indicated) |  |  | n=7  (unless otherwise indicated) | n=15 (unless otherwise indicated) | n=22 (unless otherwise indicated) |  |  | n=5 (unless otherwise indicated) | n=13 (unless otherwise indicated) | n=18  (unless otherwise indicated) |  |
|  | **Knowledge User** |  |  |  |  |  |  |  |  |  |  |  |  |  |  |
|  | **Evidence Domain** |  |  |  |  |  |  |  |  |  |  |  |  |  |  |
| 1 | Critically appraise research findings | *Very Important (71.4)* | *Important*  *(31.6)* | *Important*  *(42.3)* | **VERY IMPORTANT** |  | *Very Important (57.1)* | *Important (6.7)* | *Important*  *(22.7)* | ***Important*** |  |  |  |  |  |
| 2 | Identify and select the most appropriate evidence for the context being considered | Very Important (57.1) | Very Important  (68.4) | Very Important (65.4) |  |  | *Very Important (85.7)* | *Important/ Very Important (50.0)*  **n=14* | *Very Important*  *(61.9)*  **n=21* | ***Very Important*** |  |  |  |  |  |
| 3 | Develop research questions for literature searches | *Very Important (71.4)* | *Important (31.6)* | *Important*  *(42.3)* | **VERY IMPORTANT** |  | Important (0.0) | Important (7.1)  *n=14 | Important  (4.8)  *n=21 |  |  |  |  |  |  |
| 4 | Create and execute an efficient search strategy within relevant electronic databases | Slightly Important  (42.9) | Slightly Important  (26.3) | Slightly Important  (30.8) |  |  | Slightly Important (0.0) | Slightly Important (0.0)  *n=14 | Slightly Important  (0.0)  *n=21 |  |  |  |  |  |  |
| 5 | Keep up to date on relevant literature for the context being considered | Important  (42.9) | Important  (47.4) | Important  (46.2) |  |  | Important (28.6) | Important (28.6)  *n=14 | Important  (28.6)  *n=21 |  |  |  |  |  |  |
| 6 | Explain the different types of knowledge (e.g. research, practice, theory) that contribute to decision-making | Important  (42.9) | Important/ Very Important (50)  *n=18 | Important (48)  *n=25 |  |  | Important (0.0) | Important (14.3)  *n=14 | Important  (9.5)  *n=21 |  |  |  |  |  |  |
| 7 | Examine and interpret selected evidence from a literature search | Important (42.9) | Important  (47.4) | Important  (46.2) |  |  | Important (0.0) | Important (7.1)  *n=14 | Important  (4.8)  *n=21 |  |  |  |  |  |  |
| 8 | Use implementation resources (e.g. knowledge translation tools) to apply evidence for multiple audiences | Very Important  (85.7) | Very Important  (57.9) | Very Important  (65.4) |  |  | Very Important (71.4) | Very Important (64.3)  *n=14 | Very Important  (66.7)  *n=21 |  |  |  |  |  |  |
| 9 | Describe the research process (e.g. research question, research ethics, different methodologies, data collection and analyses) | Important  (14.3) | Important  (31.6) | Important  (26.9) |  |  | Important (14.3) | Slightly Important (0.0)  *n=13 | Slightly Important  (5.0)  *n=20 |  |  |  |  |  |  |
| 10 | Differentiate between evaluation, research, and quality improvement |  |  |  |  |  | *Very Important (85.7)* | *Slightly Important/ Important (21.4)*  **n=14* | *Important*  *(42.9)*  **n=21* | **VERY IMPORTANT** |  | *Very Important*  *(60.0)* | *Important (23.1)* | *Important*  *(33.3)* | ***Important*** |
| 11 | Utilize various resources (e.g., resource librarians) for evidence-gathering activities |  |  |  |  |  | Important (42.9) | Slightly Important (0.0)  *n=14 | Important  (14.3)  *n=21 |  |  | Important (40.0) | Slightly Important (7.7) | Slightly Important  (16.7) |  |
|  | **Teamwork Domain** |  |  |  |  |  |  |  |  |  |  |  |  |  |  |
| 1 | Help others access and appraise evidence | Important  (28.6) | Important  (42.1) | Important  (38.5) |  |  | Important (14.3) | Slightly Important/ Important (0.0)  *n=14 | Important  (4.8)  *n=21 |  |  |  |  |  |  |
| 2 | Actively seek opportunities to engage in the research process | Important  (42.9) | Important  (47.4) | Important  (46.2) |  |  | Important (14.3) | Slightly Important/ Important (0.0)  *n=14 | Important  (4.8)  *n=21 |  |  |  |  |  |  |
| 3 | Clarify the roles of individual team members |  |  |  |  |  | *Very Important (57.1)* | *Important (26.7)* | *Important*  *(36.4)* | ***Important*** |  | *Very Important*  *(60.0)* | *Important (33.3)*  **n=12* | *Important*  *(41.2)*  **n=17* | ***Important*** |
|  | **KT Activities Domain** |  |  |  |  |  |  |  |  |  |  |  |  |  |  |
| 1 | Utilize appropriate KT framework(s) for the context being considered | *Very Important*  *(71.4)* | *Important*  *(47.4)* | *Very Important*  *(53.8)* | ***Very Important*** |  | *Very Important (71.4)* | *Important (30.8)*  **n=13* | *Important*  *(45.0)*  **n=20* | **VERY IMPORTANT** |  |  |  |  |  |
| 2 | Lead the team in synthesizing research evidence to improve healthcare services | Important  (28.6) | Important  (36.8) | Important  (34.6) |  |  | Important (42.9) | Slightly Important (0.0)  *n=14 | Slightly Important  (14.3)  *n=21 |  |  |  |  |  |  |
|  |  |  |  |  |  |  |  |  |  |  |  |  |  |  |  |
|  |  | **ROUND 1** |  |  |  |  | **ROUND 2** |  |  |  |  | **ROUND 3** |  |  |  |
|  |  | Median  (% agree) | Median  (% agree) | Median  (% agree) |  |  | Median  (% agree) | Median  (% agree) | Median  (% agree) |  |  | Median  (% agree) | Median  (% agree) | Median  (% agree) |  |
|  |  | **KU** | **Researcher** | **Pooled** | **Equi-median** |  | **KU** | **Researcher** | **Pooled** | **Equi-median** |  | **KU** | **Researcher** | **Pooled** | **Equi-median** |
|  |  | n=7 (unless otherwise indicated) | n=19  (unless otherwise indicated) | n=26 (unless otherwise indicated) |  |  | n=7 (unless otherwise indicated) | n=15 (unless otherwise indicated) | n=22 (unless otherwise indicated) |  |  | n=5 (unless otherwise indicated) | n=13 (unless otherwise indicated) | n=18  (unless otherwise indicated) |  |
|  | **Researcher** |  |  |  |  |  |  |  |  |  |  |  |  |  |  |
|  | **Evidence Domain** |  |  |  |  |  |  |  |  |  |  |  |  |  |  |
| 1 | Identify and set priorities regarding policy needs and research options | *Important (28.6)* | *Very Important*  *(73.7)* | *Very Important*  *(61.5)* | ***Very Important*** |  | *Very Important (71.4)* | *Important/ Very Important (50.0)*  **n=14* | *Very Important*  *(57.1)*  **n=21* | ***Very Important*** |  |  |  |  |  |
|  | **Teamwork Domain** |  |  |  |  |  |  |  |  |  |  |  |  |  |  |
| 1 | Understand how intellectual property considerations may impact the dissemination process |  |  |  |  |  | *Very Important (71.4)* | *Important (46.7)* | *Very Important*  *(54.5)* | ***Very Important*** |  | Very Important  (80.0) | Very Important (61.5) | Very Important  (66.7) |  |
|  | **KT Activities Domain** |  |  |  |  |  |  |  |  |  |  |  |  |  |  |
| 1 | Articulate the difference between research and evaluation for a particular context | Very Important  (71.4) | Very Important  (63.2) | Very Important  (65.4) |  |  | *Very Important (71.4)* | *Important/ Very Important (50.0)*  **n=14* | *Very Important*  *(57.1)*  **n=21* | ***Very Important*** |  |  |  |  |  |
| 2 | Understand the role and practices of social media in the KT process | *Important*  *(42.9)* | *Very Important*  *(63.2)* | *Very Important*  *(57.7)* | ***Very Important*** |  | *Very Important (85.7)* | *Important (42.9)*  **n=14* | *Very Important*  *(57.1)*  **n=21* | ***Very Important*** |  |  |  |  |  |

“% Agree” refers to the percentage of participants who agreed with either “Very Important” or “Extremely Important.”

Italicized text: indicate instances where there was discordance between KU and researcher group median ratings (i.e., for one group median was “Very Important” or above, and for the other group the median was below “Very Important”)

Bolded and Italicized text: signify that the equimedian rating is concordant with the pooled median rating for that competency

Bolded and capitalized text: signify that the equimedian rating is discordant with the pooled median rating for that competency
